# *Salmonella* Interacts With Autophagy to Offense or Defense

**DOI:** 10.3389/fmicb.2020.00721

**Published:** 2020-04-22

**Authors:** Shu Wu, Yiru Shen, Shan Zhang, Yunqi Xiao, Shourong Shi

**Affiliations:** ^1^Department of Feed and Nutrition, Poultry Institute, Chinese Academy of Agricultural Sciences, Yangzhou, China; ^2^Institute of Effective Evaluation of Feed and Feed Additive (Poultry institute), Ministry of Agriculture, Yangzhou, China; ^3^College of Animal Science and Technology, Hunan Agricultural University, Changsha, China; ^4^Jiangsu Co-Innovation Center for Prevention and Control of Important Animal Infectious Diseases and Zoonoses, Yangzhou University, Yangzhou, China

**Keywords:** *Salmonella*, autophagy, xenophagy, autophagy adaptor, drug regulation

## Abstract

Autophagy is an important component of the innate immune system in mammals. Low levels of basic autophagy are sustained in normal cells, to help with the clearance of aging organelles and misfolded proteins, thus maintaining their structural and functional stability. However, when cells are faced with challenges, such as starvation or pathogenic infection, their level of autophagy increases significantly. *Salmonella* is a facultative intracellular pathogen, which imposes an economic burden on the poultry farming industry and human public health. Previous studies have shown that *Salmonella* can induce the autophagy of cells following invasion, which to a certain extent helps to protect the cells from bacterial colonization. This review summarizes the latest research in the field of *Salmonella*-induced autophagy, including: (i) the autophagy induction and escape mechanisms employed by *Salmonella* during the infection of host cells; (ii) the effect of autophagy on intracellular *Salmonella*; (iii) the important autophagy adaptors that recognize intracellular *Salmonella* in host cells; and (iv) the effect of autophagy-modulating drugs on *Salmonella* infection.

## Introduction

**FIGURE 1:**
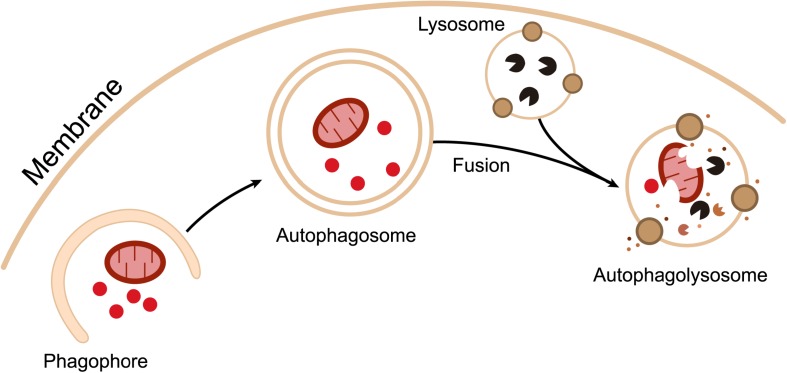
Outline of autophagic flux. Intracellular components are surrounded by cup-shaped double-membraned structures called “phagophores.” Phagophores gradually extend and eventually completely engulf the captured substrates to form double-membraned “autophagosomes.” Autophagosomes then fuse with lysosomes to form “autophagolysosomes,” where degradation occurs.

The concept of autophagy was first proposed by de Duve in 1963, to describe the process that transports cytoplasmic protein aggregates or damaged organelles to the lysosome for degradation and recycling (Yang and Klionsky, 2010). It is an evolutionarily conserved essential intracellular eukaryotic cellular process, mediated by monolayer or bilayer-containing membrane vesicles called “autophagosomes,” Generally, autophagy can be subdivided into three types according to the distinct delivering processes of substrates to lysosomes: macroautophagy, microautophagy, and chaperone-mediated autophagy (CMA) (Xie and Klionsky, 2007). Macroautophagy uses “phagophore,” a horseshoe-shaped double-membraned structure, to envelop the captured cargoes and finally completely swallow them, forming a double-membraned autophagosome. By contrast, microautophagy and CMA usually form monolayer autophagosomes through direct or unfolded chaperone proteins mediated uptake of cargoes at the lysosome surface. Although the autophagosomes themselves are not degradative by nature, the subsequent fusion of autophagosomes with lysosomes provides a closed environment for degradation (Casanova, 2017). The term “autophagic flux” is used to describe the dynamic process of autophagosome synthesis, delivery of autophagic substrates to the lysosome, and degradation of autophagic substrates inside the lysosome (Figure 1; Mizushima et al., 2010). In the last 20 years, over 30 autophagy-related (Atg) proteins have been identified to involve in autophagic flux (Xie and Klionsky, 2007; Casanova, 2017). Autophagy plays an important role in maintaining protein metabolism and the stability of the cellular environment. Any part of autophagy flux being blocked will lead to autophagy dysfunction and corresponding consequences. Cellular autophagic activity is usually low under homeostatic conditions, but can be markedly up-regulated by numerous stimuli, such as nutrient starvation, hypoxia, energy loss, or microbial infection (Mizushima et al., 2010). Autophagy can be non-selective or selective. Non-selective autophagy displays no specificity toward the cargo, randomly capturing cytoplasmic components for degradation. Selective autophagy specifically degrades various cargoes, including organelle-specific autophagy (mitophagy, pexophagy, reticulophagy, etc.) and xenophagy (which is the degradation of microorganisms) (Cui et al., 2019).

Xenophagy serves as an important innate immune mechanism for the intracellular clearance of pathogenic bacteria. Many invasive bacterial pathogens, such as *Salmonella*, *Shigella*, *Listeria*, *Legionella*, *Mycobacterium*, *Franciscella*, and Group A *Streptococcus* induce autophagy (Casanova, 2017). However, some pathogens have evolved complex escape mechanisms that either inhibit autophagy entirely or evade recognition and capture via autophagy mechanisms (Huang and Brumell, 2014; Sorbara and Girardin, 2015; Kohler and Roy, 2017). In fact, studies of *Listeria, Shigella, Mycobacterium*, and *Salmonella* have shown that xenophagy plays an important role in limiting intracellular bacterial replication (Cemma and Brumell, 2012). Of these, *Salmonella* autophagy currently represents the hottest research topic in the field of xenophagy. This was demonstrated by results from a PubMed search for papers, containing the words “autophagy” within the title/abstract and “bacteria” within the main body of text, published prior to September 2019. In total, we found 356 research papers, of which 86 were related to *Salmonella* autophagy, accounting for roughly 1/4 of all manuscripts. Therefore, this article will summarize the recent research progress in the area of *Salmonella* autophagy.

## Manipulation of Host Cell-Mediated Autophagy by *Salmonella*

Pathogenic *Salmonella* harbors two type III secretion systems (T3SS1 and T3SS2), which are encoded by two different Salmonella pathogenicity islands; SPI-1 and SPI-2, respectively (Hansen-Wester and Hensel, 2001). These T3SSs are needle-like protein complexes that penetrate host cell membranes and inject various bacterial effectors into the cytoplasm (Xie and Klionsky, 2007). T3SS1 expression is essential for bacterial host cell invasion, while T3SS2 expression is induced only after entering host cells, and is required for intracellular bacterial survival (Figueira and Holden, 2003; Figueira et al., 2013).

T3SS1 and T3SS2 mediate the translocation of a large number of bacterial effectors. After invasion, some of these T3SS effectors can hijack the host cell endocytic pathway and convert the phagosomes into monolayer vacuoles, known as “*Salmonella*-containing vacuoles” (SCVs), which are well suited to the survival and replication of *Salmonella* (Steele-Mortimer, 2008). A recent study has shown that the expression of T3SS1 and its effectors were significantly down-regulated 1–2 h post-invasion, while the expression of T3SS2 increased with time, accompanied by the maturation of SCVs. The expression of these two groups of effectors therefore plays a synergistic role in the modification and maturation of SCVs during the infection of epithelial cells with *Salmonella* (Hautefort et al., 2008).

### *Salmonella* Induces Autophagy

*Salmonella* invades host epithelial cells actively and partially triggers autophagy via the T3SS1-mediated disruption of membrane integrity and the subsequent translocation of effector proteins into cell. Pores formed by T3SS1 result in the leakage of amino acids from the cell, which leads to acute starvation stress. Amino acid starvation inhibits the activity of mTORC1 through a complex signaling cascade, activates the EIF2AK4/GCN2-EIF2S1/eIF2α/ATF3 pathway, and ultimately induces autophagy (Tattoli et al., 2012a, b).

**FIGURE 2:**
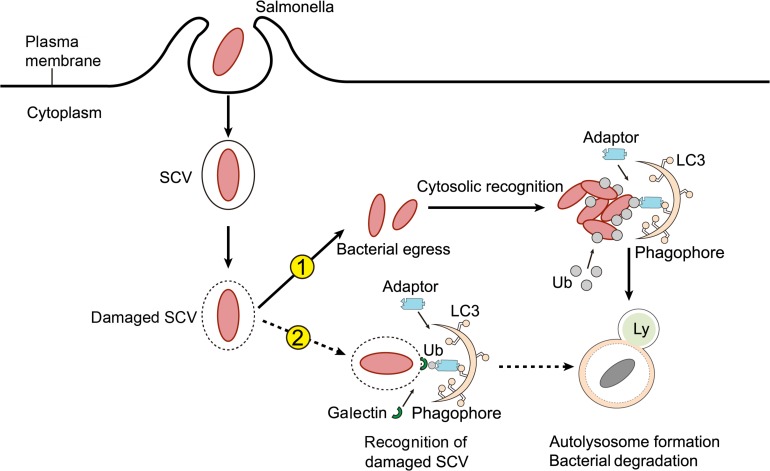
How *Salmonella* induces autophagy. After internalization by host cells, *Salmonella* resides within a modified phagosomal compartment called the *Salmonella*-containing vacuole (SCV). While the needle-like Type III secretion system (T3SS-1) of *Salmonella* can cause damage to the SCV, resulting in one of two consequences for the pathogen. Firstly, if the T3SS-mediated damage is sufficient, the SCV can rupture, allowing bacteria to enter the cytoplasm. Cytosolic *Salmonella* are rapidly tagged with ubiquitin (Ub) and marked for autophagy by adaptor molecules. Autophagy adaptors interact with Atg8 family members, such as LC3, to promote autophagosome biogenesis. Secondly, *Salmonella* residing in damaged SCVs can also be targeted for autophagy. In this case, modified carbohydrate structures on the SCV recruit galectins, adaptors, and ubiquitin to the membrane, which tag these compartments for autophagic elimination.

*Salmonella* can induce autophagy via two pathways (Figure 2). The first involves the recognition of intracellular *Salmonella* by ubiquitin. As mentioned above, intracellular *Salmonella* mainly resides in SCVs, surviving and replicating in these membrane-bound compartments (Malik-Kale et al., 2012). However, a small number of *Salmonella* can penetrate the SCV monolayer through the use of their needle-like T3SS1, and escape from the SCV into the cytoplasm to achieve high rates of replication. High copy numbers of intracellular *Salmonella* are quickly recognized by the ubiquitination system of host cells, resulting in the formation of an intensive ubiquitin chain layer surrounding the bacteria. Ubiquitinated *Salmonella* is recognized by autophagy adaptors such as NDP52, OPTN, and p62. These adaptors bind to the ubiquitinated bacteria via their ubiquitin binding domain and interact with the autophagic membrane anchoring protein LC3 to direct the bacteria to primary autophagosomes (Mostowy et al., 2011). The second strategy that *Salmonella* employs to induce autophagy involves the recognition of destroyed SCVs resulting from the pore-forming properties of T3SS1. In this scenario, *Salmonella* need not be in a planktonic state for autophagic capture and degradation (Birmingham and Brumell, 2006). As previously mentioned, the expression of T3SS1 continues for 1–2 h after bacterial internalization. In the meantime, T3SS1 mediates SCV membrane destruction in the same way that it breaks down the cell membrane (Kreibich et al., 2015). If the damage is sufficiently substantial, the SCV lumen will become accessible to the cytoplasmic ubiquitin ligases, labeling *Salmonella* by ubiquitination (Perrin et al., 2004). In addition to ubiquitination, SCV damage is also recognized by galectin-8, a cytoplasmic galactose lectin. Galectin-8 serves as a monitor for endolysosomal integrity by recognizing β-galactoside, which is normally present on the inner surface of SCVs. SCVs identified by galectin-8, in turn, recruit autophagy adaptors to the SCV membrane, thereby attaching damaged SCVs to primary autophagosomes (Thurston et al., 2012).

### The Principal Effectors of *Salmonella*-Specific Autophagy Induction

As mentioned above, *Salmonella* can induce host cell-mediated autophagy. We will now discuss in more detail which *Salmonella* effector proteins are involved in autophagy induction. To date, studies have found that *Salmonella* cytolethal distending toxin B (cdtB), asparaginase, SipB virulence protein, and β-barrel outer membrane protein (β-OMP) are involved in the induction of *Salmonella* autophagy (Table 1).

**TABLE 1:** Mechanisms used by *Salmonella* to induce or escape autophagy.

Bacterium	Bacterial factors	Model systems	Refs
Inducing autophagy:			
*S.* Javiana	Cytolethal distending toxin B (CdtB)	J774A.1	25
*S.* Typhimurium	Type II L-asparaginase	T cells	26
*S.* Typhimurium	SipD	Bone marrow-derived primary macrophages	29
*S.* Typhimurium	β-barrel outer membrane protein (β-OMP)	THP1 and Caco-2/TC7 cells	30
Escaping autophagy:			
*S.* Typhimurium	SsrB, ssaV	Bone marrow-derived primary macrophages	33
*S.* Typhimurium	SseG, SseF	HEK293T, HeLa, THP-1, RAW264.7, mice	36
*S.* Typhimurium	SseL	HeLa, RAW264.7, bone marrow-derived primary macrophages	38
*S.* Typhimurium	PR ST98 plasmid	THP-1, J774A.1, embryonic fibroblasts	40–43
*S.* Typhimurium	SpvB	J774A.1, HeLa, mice, zebrafish	46–48
*S.* Typhimurium	reactive persulfides	RAW264.7, bone marrow-derived primary macrophages	52
*S.* Typhimurium	–	FAK^–/–^ peritoneal macrophages, mice lacking macrophage-specific FAK	54

The cytolethal distending toxin (CDT), a type of toxin which plays an important role in bacterial pathogenesis, was initially found to be produced by *Escherichia coli* (*E. coli*), and later shown to be a product of many types of Gram-negative bacteria. CDT mimics the role of DNA enzymes and damages macrophage DNA, thus causing cell cycle arrest and macrophage swelling and apoptosis (Hassane et al., 2003). CDT consists of three polypeptides: cdtA, cdtB, and cdtC. Of these, cdtA and cdtC transport cdtB into host cells, while cdtB mediates cellular chromosomal DNA degradation (Lara-Tejero and Galan, 2001). *Salmonella* expresses cdtB, but not cdtA and cdtC. Williams et al. (2015) found that macrophages infected with the *Salmonella* Javiana (SJ) mutant strain lacking cdtB, displayed lower levels of LC3 and autophagy-related gene expression than cells infected with the wild-type strain, suggesting that cdtB was involved in the SJ-mediated autophagy induction in host cells.

L-asparaginase (L-ASNase) is a hydrolase that converts L-asparagine to L-aspartic acid and ammonia. L-ASNase has anti-tumor activity and is widely used in the fields of medicine and nutrition. Torres et al. (2016) found that *Salmonella* can produce a type II L-ASNase, which can hydrolyze the L-asparagine required for T cell activation, thereby inhibiting mTOR signaling and activating autophagy.

The simplest virulence strategy employed by *Salmonella* is its ability to penetrate the intestinal mucosal barrier. *Salmonella* invasive protein D (SipD) is an effector protein located within SPI-1. SipD is secreted primary by T3SS-1 and binds to the top of the T3SS-1 structure. During the invasion stage of infection, *Salmonella* makes contact with the endothelial cell membrane via the effector SipD (Lunelli et al., 2011; Rathinavelan et al., 2014). Hernandez et al. (2003) showed that the infection of macrophages by SipD-deficient *Salmonella* did not result in autophagy, in contrast to wild type *Salmonella*.

Gram-negative bacteria and the mitochondrial outer membrane possess a unique β-barrel tertiary structure, which can serve as a pathogen-associated molecular pattern (PAMP) for innate immune system recognition. Chaudhary et al. (2018) found that the β-OMP purified from *Salmonella*, induced endosomal acidification, LC3B lipidation, and p62 degradation, in addition to reducing mTORC2 and Akt phosphorylation, suggesting that β-OMP is also an important effector protein of *Salmonella*-induced host cell-mediated autophagy.

### The Escape Mechanism Employed by *Salmonella* to Evade Host Cell Autophagy

It is well known that autophagy, as a catabolic pathway for the degradation of dysfunctional cellular proteins, organelles, and pathogenic microorganisms, plays an important role in host defense, and is an integral part of innate and adaptive immune responses. *Salmonella*, as a typical facultative intracellular bacterium, can induce the autophagy of host cells following infection, and maintain normal physiological functions by degrading pathogens through autophagy. On the other hand, *Salmonella* has evolved a unique escape mechanism to evade autophagy, which can interfere with the process of autophagy-mediated pathogen degradation and clearance, allowing *Salmonella* to persist inside host cells. *Salmonella*-induced autophagy is in fact temporary, occurring mainly in the early stages of infection. It has been demonstrated that, in cultured epithelial cells, autophagy peaks 1 h following infection initiation and lasts for only 3 h post-infection (Tattoli et al., 2012b), coinciding with the time period involved in the up- or down-regulation of the two T3SSs. Down-regulation of T3SS1 and up-regulation of T3SS2 may lead to the normalization of plasma membrane integrity and amino acid levels, thus inhibiting autophagy. Previous studies have also confirmed that the induction of T3SS2 and its effectors plays a crucial role in the mechanism employed by *Salmonella* to escape phagocytosis. In addition, *Salmonella* virulence plasmids and genes, as well as bacterial sulfur metabolites, are also involved in its evasion of autophagy.

SsrB, a member of the NarL/FixJ subfamily of DNA-binding response regulators, specifically regulates the majority of the SPI-2-encoded virulence factors, and is mainly expressed after SCV acidification (Walthers et al., 2007). SsaV is also a member of T3SS2 that injects SPI-2 virulence factors into host cells (Grant et al., 2012). Ganesan et al. (2017) found that the *Salmonella* virulence factors SsrB and SsaV disrupted Sirt1/LKB1/AMPK signaling, thus activating mTOR and evading autophagy; it was demonstrated for the first time that the Sirt1/LKB1/AMPK complex was the target of effectors encoded by *Salmonella* SPI-2. *Salmonella* therefore manipulates mTOR activity through the actions of the virulence factors SsrB and SsaV, thus evading the defense mechanism of autophagic host cells.

SseG and SseF are two effector proteins encoded by SPI-2 that play a crucial role in the process of bacterial infection and the maintenance of SCV membrane integrity (Kuhle and Hensel, 2002; Deiwick et al., 2006). Feng et al. (2018) found that the SseF and SseG secreted by *Salmonella* Typhimurium could impair the recruitment and activation of ULK1 by blocking Rab1A activity in host cells. This resulted in a reduction in PI3P production, ultimately impeding autophagosome formation in host cells, and thereby establishing a replicative niche for the bacteria in the cytoplasm. Furthermore, the survival and growth rates of SseF- or SseG-deficient *Salmonella* strains were reduced both *in vitro* and *in vivo*, but could be restored to wild type levels by depleting Rab1A. These findings suggest that the virulence factors SseG and SseF can help *Salmonella* evade autophagy and the host defense system via the inactivation of GTPase Rab1A.

SseL is another SPI-2 T3SS-encoded effector protein, and an important *Salmonella* virulence factor, which regulates the host’s inflammatory response by deubiquitization (Le Negrate et al., 2008). As mentioned previously, bacterial infection leads to the formation of ubiquitin aggregates in cells, which are recognized by autophagy adaptors before entering the autophagic degradation pathway. Mesquita et al. (2012) found that the ubiquitination markers of *Salmonella*-infected cells could be counteracted by the deubiquitinase SseL, thereby impeding their autophagic degradation and favoring intracellular bacterial replication. The results showed that SseL was also important in enabling *Salmonella* to escape autophagic degradation. However, there are some differences in the genetic sequences encoding SseL between *Salmonella* Typhimurium and *Salmonella* Enteritidis, implying that the functions of SseL may also vary between different *Salmonella* strains and even different serotypes of *Salmonella* Enteritidis.

The pR (ST98) plasmid is a conjugative transfer plasmid that mediates the drug resistance and virulence of *Salmonella* Typhi. pR (ST98) has a molecular weight of 98.6 × 10^6^ Da (about 159 kb), and was first identified and reported by Huang et al. (2005). Subsequent studies in their laboratory have shown that mutant *Salmonella*, harboring a pR (ST98) plasmid deletion, inhibited autophagy in infected macrophages (He et al., 2012; Chu et al., 2014; Wu et al., 2014) and mouse embryonic fibroblasts (Lv et al., 2012), while enhancing *Salmonella* proliferation within cells and consequently promoting cell death. These results demonstrate that the pR (ST98) plasmid enables *Salmonella* to escape autophagy.

The *Salmonella* plasmid virulence (*spv*) locus is a highly conserved sequence commonly carried by all pathogenic *Salmonella*. It can increase the growth of *Salmonella* in extraintestinal tissue cells, and is associated with bacterial serum resistance, adhesion, and colonization (Kuźmińska-Bajor et al., 2012). The *spv* region contains five genes: *spvR, spvA, spvB, spvC*, and *spvD*. Of these, *spvB* is a structural gene, which has a great influence on bacterial virulence (Guiney and Fierer, 2011). It has been found that *spvB* can depolymerize actin, inhibit autophagy in human epithelial HeLa cells, macrophage-like J774A.1 cells, and BALB/c mice, and aggravate the host’s inflammatory damage to *Salmonella* infection (Chu et al., 2016). Moreover, in the zebrafish model infected with *Salmonella*, it was also confirmed that *Salmonella spvB* could promote bacterial survival and intestinal damage by inhibiting autophagy (Li et al., 2016; Wu et al., 2016). Recent work using the Raw264.7 mouse macrophage cell line and zebrafish larvae as models, also showed that the *Salmonella spv* locus could inhibit the type I interferon response and the chemotaxis of neutrophils by suppressing autophagy (Wang et al., 2019). In conclusion, these data indicate that *spvB* is an important virulence factor employed by *Salmonella* to escape autophagy.

In addition, *Salmonella* and other pathogenic bacteria produce reactive persulfides, such as cysteine persulfate (CysSSH) and glutathione persulfate (GSSH), during their metabolism of sulfur (Ida et al., 2014; Akaike et al., 2017). It has been shown that the reactive persulfides produced by *Salmonella* can metabolize 8-nitro-cGMP to regulate macrophage autophagy (Shahzada et al., 2018). 8-nitro-cGMP signaling is necessary for infected macrophages to clear intracellular *Salmonella* by autophagy, suggesting that bacteria-derived reactive persulfides can also help *Salmonella* escape from autophagic degradation (Owen et al., 2007).

Although autophagic escape mechanisms are predominantly orchestrated by bacterial factors, the focal adhesion kinase (FAK), a non-receptor tyrosine kinase known for its role in adhesion-mediated signal transduction in a variety of cell types (Owen et al., 2014), was also found to be implicated. FAK is recruited to the SCV surface via the *Salmonella* SPI-2 system, leading to the amplification of signals through the Akt-mTOR axis, thus inhibiting autophagy and promoting the survival of bacteria within macrophages. Indeed, FAK^–/–^ macrophages display a weaker Akt/mTOR signal, resulting in the improved autophagic capture of bacteria, and ultimately reducing overall bacterial survival. *In vivo*, knocking out FAK in macrophages also results in the rapid elimination of *Salmonella* from various tissues. Collectively, these results suggest that FAK represents a means for *Salmonella* to escape autophagy through T3SS2, thereby promoting its viability in cells (Owen et al., 2014).

## The Effect of Autophagy on Intracellular *Salmonella*

At present, the consensus regarding the effect of autophagy on intracellular *Salmonella* is that autophagy helps cells to remove invasive intracellular bacteria. However, in recent years, it has been reported that *Salmonella* can also exploit autophagy to promote bacterial replication.

### Autophagy Promotes the Elimination of *Salmonella* From Host Cells

In 2006, Birmingham et al. observed that some intracellular *Salmonella* are recognized by autophagy under *in vitro* infection conditions, and confirmed that autophagic recognition restricted the replication of intracellular *Salmonella*. To further delineate the role of autophagy in limiting the growth of intracellular *Salmonella*, Birmingham and his colleagues utilized Atg5^–/–^ mouse embryonic fibroblasts (MEFs) infected with *Salmonella* Typhimurium to measure the intracellular growth of *Salmonella*. The results showed that, compared with wild type MEFs, the growth of bacteria in Atg5^–/–^MEF cells increased. These results suggest that the autophagy induced by *Salmonella* infection enables host cells to restrict the growth of bacteria and protect their cytoplasm from bacterial colonization (Birmingham et al., 2006). Since then, other studies have strengthened this concept. Jia et al. (2009) studied the effect of autophagy gene inactivation on *Salmonella* infection in two model organisms, *Cryptorhabditis elegans* and *Reticulium discoides*. The results showed that genetic inactivation of the autophagy pathway in both organisms resulted in increased bacterial intracellular replication and decreased animal lifespan, suggesting that autophagy plays an important role in the host defense against intracellular *Salmonella in vivo*. Conway et al. (2013) generated mice whose epithelial cells were deficient in the autophagy-implicated protein Atg16L1. After the mice were infected with *Salmonella* Typhimurium, the autophagy level, immune function, and bacterial clearance ability of the cecum and small intestine tissues were analyzed. The results showed that *Salmonella* infection increased autophagy within the intestinal epithelium in control mice but made no difference to the level of autophagy observed in the epithelial cells of ATG16L1-deficient animals. Furthermore, mice deficient in ATG16L1 displayed abnormal immune function and decreased bacterial clearance ability, compared to the control mice. It was suggested that the autophagy performed by intestinal epithelial cells helps mice to eliminate infected bacteria and prevent systemic infection. Curt et al. (2014) used the *C. elegans* model to demonstrate that inhibition of the autophagy gene *bec1* in intestinal epithelial cells made the organism more susceptible to *Salmonella* infection, suggesting that autophagy promoted the elimination of *Salmonella* by intestinal epithelial cells. ATP6V0D2 is a critical component mediating autophagosome-lysosome fusion in macrophage cells. *Atp6v0d2*-deficient macrophage cells exhibited decreased levels of *Salmonella* Typhimurium elimination, while *Atp6v0d2*-knockout mice became more susceptible to *Salmonella* Typhimurium-induced death (Xia et al., 2019). Raw264.7 cells pretreated with the autophagy inhibitors 5Z-7-oxozeaenol or compound C, prior to infection with *Salmonella* Typhimurium, also showed accelerated intracellular *Salmonella* replication (Liu et al., 2019).

### Cell Autophagy Facilitates *Salmonella* Survival in Host Cells

As an important component of the host’s defense system, autophagy is recognized for its role in degrading *Salmonella* and limiting its replication and proliferation. Contrary to this concept, however, autophagy has recently been reported to facilitate the replication of *Salmonella* in HeLa cells. Yu et al. (2014) investigated the density of intracellular *Salmonella* and found that the *Salmonella* targeted by autophagy mainly resided in the cytosol and replicated rapidly; while, the bacteria localized to intact SCVs were not associated with autophagosomes and replicated slowly. In addition, depletion of autophagy mediators by specific siRNAs (p62/LC3/Atg5/Atg16L1/TBK1) significantly inhibited the proliferation of *Salmonella* in the cytoplasm of HeLa cells. High rates of autophagy-targeted *Salmonella* replication in the cytoplasm eventually lead to increased cell exfoliation, promoting the spread of *Salmonella* to adjacent cells. Researchers speculate that the variation in results could be attributed to the off-target effects of high-dose siRNAs used by other groups or the incomplete dissociation of membrane-associated *Salmonella* when quantifying intracellular bacterial numbers. The cell type and time point chosen for the assessment of bacterial replication may also have an impact (Yu et al., 2014). Interestingly, prior to their report, it was also shown that knocking out Rab1, a GTPase required for *Salmonella* autophagy, reduced the rate of *Salmonella* replication in HeLa cells (Huang et al., 2011). Although the authors initially speculated that this result was due to the inhibition of bacterial replication in SCVs, it was later confirmed by live imaging that the replicative niche for *Salmonella* was predominantly the cytoplasm (Malik-Kale et al., 2012). Therefore, the inhibition of bacterial replication caused by the deletion of Rab1 can also be attributed to the inhibition of autophagy, thus reducing *Salmonella* replication in the cytoplasm.

## Adaptors of *Salmonella* Autophagy

**FIGURE 3:**
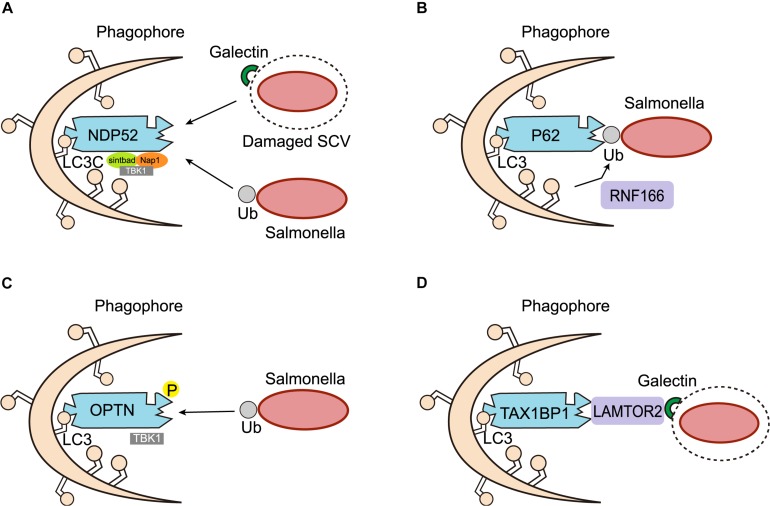
Adaptors of *Salmonella* autophagy. **(A)** NDP52 functions as a scaffold for the assembly of a TBK1-Sintbad-Nap1 signaling complex, transports *Salmonella* marked by ubiquitin (Ub) or damaged SCV membranes marked by galectin-8 into the autophagic pathway through an interaction with LC3C. **(B)** P62 serves as an autophagy adaptor, was recruited to ubiquitinated *Salmonella* targeted by autophagy. The ring finger protein 166 (RNF166) is vital for the recruitment of the autophagy adaptor p62 to ubiquitinated *Salmonella*. **(C)** OPTN recognizes ubiquitinated *Salmonella* and targets them for autophagy. Phosphorylation (P) of OPTN by TBK1 promotes its interaction with LC3. **(D)** TAX1BP1, a close homolog of NDP52, also acts as an autophagy adaptor in *Salmonella* autophagy. In response to membrane damage caused by bacteria, LAMTOR2 is recruited to the damaged membrane and associates with galectins. LAMTOR2 then recruits TAX1BP1 to promote the formation of autophagy.

In accordance with the autophagy of other bacterial species, intracellular *Salmonella* labeled with E3 ubiquitin ligase also needs to be recognized by autophagy adaptors in order to be targeted for autophagic degradation. Autophagy adaptors are proteins that simultaneously interact with specific substrates and components of the autophagy machinery, and serve to link substrate degradation to newly formed autophagosomes. Autophagy adaptors generally possess several protein-protein interaction domains, such as the LC3 interaction region (LIR) for LC3 binding, and the ubiquitin-associated (UBA) domain for interacting with ubiquitin-coated substrates. Researchers have found four main autophagy adaptors implicated in *Salmonella* autophagy, namely NDP52, p62, OPTN, and TAX1BP1 (Figure 3).

NDP52 is an important adaptor of selective autophagy. It maintains cellular homeostasis mainly through a process called “mitophagy,” which removes damaged mitochondria from cells (Heo et al., 2015). Several studies have also proven that NDP52 plays a crucial role in the process of xenophagy. For instance, various bacteria, such as *Salmonella*, *Shigella flexneri*, and *Streptococcus pyogenes*, have been shown to be targets of NDP52-mediated selective autophagy (von Muhlinen et al., 2010; Mostowy et al., 2011; Minowa-Nozawa et al., 2017). In the context of *Salmonella* autophagy, NDP52 acts as a bridging junction to specifically recognize ubiquitinated intracellular *Salmonella*. It was found that NDP52 selectively binds LC3C through its non-typical LIR and transports ubiquitinated *Salmonella* into the autophagic pathway. Cells lacking either NDP52 or LC3C therefore cannot protect their cytoplasm against *Salmonella* infection by antibacterial autophagy (von Muhlinen et al., 2012, 2013). Meanwhile, NDP52 also recruits TANK-binding kinase 1 (TBK1), an IKK family kinase that coexists with NDP52 on bacterial surfaces, by combining Nap1 and Sintbad, to interact with LC3. Deletion of NDP52 and TBK1, increases the proliferation of intracellular *Salmonella*, and causes the ubiquitin-modified bacteria to aggregate in ATG8/LC3(+) autophagosomes. These results suggest that NDP52 and the ubiquitin system can be used to activate autophagy and combat *Salmonella* colonization of the cytoplasm of human cells (Thurston et al., 2009; von Muhlinen et al., 2010). Although NDP52 is known to be important for the selective autophagic degradation of invasive pathogens, the mechanism underlying its recognition of ubiquitinated bacteria remains unclear. Through biochemical and structural analysis, Xie et al. (2015) found that the C-terminal cargo recognition region of NDP52 contains a typical C_2_H_2_-type zinc finger, which can specifically bind to cargo-covered ubiquitin or ubiquitin chains, revealing a new recognition and targeting mechanism of ubiquitin-coated substrates by the ubiquitin-binding autophagy adaptor NDP52 in selective autophagy. In addition to serving as an adaptor of xenophagy, targeting ubiquitinated bacteria for primary autophagy *in vitro*, NDP52 also promotes the maturation of autophagosomes containing *Salmonella*. These two unrelated roles depend on the different NDP52-binding domains and chaperone proteins, respectively. Specifically, NDP52 promotes autophagy maturation by interacting with LC3A, LC3B, and/or GABARAPL2 via a unique LIR and myosin VI, while the NDP52-mediated targeting of intracellular *Salmonella* for autophagy depends on its interaction with LC3C (Ivanov and Roy, 2009; Verlhac et al., 2015a, b,c). In addition to ubiquitin, galectin-8 binding to damaged SCV membranes can also recruit NDP52 to activate antimicrobial autophagy (Thurston et al., 2012; Li et al., 2013; Kwon and Song, 2018; Boyle et al., 2019; Ravenhill et al., 2019). However, the galectin-8-dependent recruitment of NDP52 to SCVs is only temporary, and therefore performs a secondary role to the ubiquitin-dependent recruitment of NPD52 (Thurston et al., 2012).

P62 is one of the earliest autophagy adaptors to have been identified and described in mammals. The role of p62 in the induction of xenophagy has mainly been investigated in the context of *Salmonella* infection. Researchers found that p62 was recruited to *Salmonella* targeted for autophagy, and that the recruitment of p62 was required for the efficient elimination of *Salmonella* by xenophagy (Zheng et al., 2009). Ishimura et al. (2014) also confirmed that once a cell was infected with *Salmonella*, p62 assembled on the pathogen and induced xenophagy. Regulators of p62 recruitment by *Salmonella* autophagy have also been reported. Heath et al. (2016) screened and identified a series of E3 ligases, of which, the ring finger protein 166 (RNF166) was found to be vital for the interaction with the autophagy network and the recruitment of the autophagy adaptor p62 to ubiquitinated *Salmonella*. Mechanistic studies have also demonstrated that RNF166 catalyzes the polyubiquitination of p62.

Optineurin (OPTN) is a 67 kDa intracellular protein distributed among different tissues, where it serves as an important autophagy adaptor involved in different types of autophagy (Wild et al., 2011; Heo et al., 2015; Richter et al., 2016; Slowicka and van Loo, 2018). Studies have proven that OPTN is necessary for limiting the proliferation of *Salmonella* following infection. Indeed, silencing OPTN weakens *Salmonella* autophagy, leading to an increase in intracellular bacterial replication (Wild et al., 2011). However, in contrast to other autophagy adaptors, the LIR of OPTN binds LC3 with very low affinity and requires input from TBK1. TBK1 can directly bind and phosphorylate OPTN on Ser^177^, enhancing its association with LC3, therefore allowing OPTN to recognize ubiquitin-coated intracellular microbes and target them for autophagy (Weidberg and Elazar, 2011; Rogov et al., 2013). To evaluate the *in vivo* importance of OPTN in inflammation and infection, Slowicka et al. (2016) generated OPTN-deficient mice, and found that they were more sensitive to *Salmonella* infection, demonstrating that OPTN in critical for bacterial clearance *in vivo*.

TAX1BP1, a close homolog of NDP52, acts as an autophagy adaptor mainly in xenophagy. The role of TAX1BP1 in xenophagy was first emphasized by a study that revealed its function in the eradication of *Salmonella* Typhimurium (Tumbarello et al., 2015). The clearance of intracellular *Salmonella* relies on the association between TAX1BP1 and motor myosin VI, which aids the fusion of autophagosomes with lysosomes. It has been demonstrated that TAX1BP1-deficiency increased the number of ubiquitin-labeled *Salmonella*, whereas the absence of myosin VI raised the number of ubiquitin- and LC3-positive *Salmonella* (Tumbarello et al., 2015). A recent study has also reported the involvement of TAX1BP1 in the clearance of *Salmonella* Typhimurium. The authors found that knocking out TAX1BP1 resulted in a decrease in the formation of autolysosomes and subsequent microbe elimination. In addition, they identified LAMTOR2 and LAMTOR1 as previously overlooked xenophagy regulators of TAX1BP1 in response to *Salmonella* infection, and confirmed that LAMTOR2 was recruited to damaged SCVs via LAMTOR1, an endosome-resident protein. LAMTOR2 was critical for the subsequent recruitment of TAX1BP1, thus accelerating the formation of autolysosomes during bacterial infection (Lin et al., 2019).

Although the above autophagic adaptors all target *Salmonella* for autophagy, it should be noted that these adaptors are independently recruited to bacteria-associated microdomains. A recent study has shown that p62 and NDP52 were recruited to *Salmonella* independently of each other, at two non-overlapping microdomains. Moreover, although antibacterial autophagy was impaired by the loss of either adaptor, there was no synergistic impairment of xenophagy when losing both adaptors simultaneously, suggesting that p62 and NDP52 may be implicated in the same pathway (Cemma et al., 2011).

## Certain Drugs Regulate Xenophagy to Combat *Salmonella* Infection

*Salmonella* represents one of the major foodborne pathogens worldwide. As a common zoonotic pathogen, *Salmonella* has a serious impact on animal husbandry, human health, and food safety. However, effective methods for the elimination of *Salmonella* infection are still limited. The main prevention and control measures for *Salmonella* infection include antibiotic therapy, competitive inhibition, and vertical purification. However, with the exception of antibiotics, the majority of antibacterial methods remain ineffective. What is worse, antibiotic therapy is also confronted with problems such as the accumulation of drug residues, drug resistance, and poor intracellular bactericidal efficiency. Therefore, there is an urgent need to develop effective agents for the control of *Salmonella* infection.

As a major constituent of innate immunity, xenophagy plays an important role in the body’s defense against foreign pathogenic bacteria. In recent years, accumulating studies have tried to find effective xenophagy-implicated agents to target *Salmonella* replication (Table 2). It is well documented that many phenolic compounds, such as Carvacrol (Gaio et al., 2017), Thymol (Marchese et al., 2016), Quercetin (Wang et al., 2018), Resveratrol (Hwang and Lim, 2015), Acacetin (Bi et al., 2016), and Epigallocatechin gallate (Nakasone et al., 2017) exhibit antibacterial activity. Recently, Al Azzaz et al. (2019) found that Resveratrol promotes the autophagy-dependent elimination of intracellular *Salmonella* both *in vitro* and *in vivo*. Ammanathan et al. (2019) found that Acacetin (5,7-dihydroxy-4-methoxyflavone), a potent xenophagy inducer, could enhance the host response against intracellular *Salmonella*. Moreover, biochanin A (BCA), a polyphenolic compound found in certain species of plants, has been proven to target *Salmonella* infection through AMPK/ULK1/mTOR-mediated autophagy, extracellular traps, and the reversal of SPI-1-dependent macrophage M2 polarization (Zhao et al., 2018). In addition, Huang (2016) found that 1,25-dihydroxyvitamin D3 may promote the autophagic removal of intracellular *Salmonella* and modulate inflammatory responses to prevent the host from the adverse effects of excessive inflammation. Kim et al. (2018) evaluated the antibacterial effect of bioprocessed (fermented) rice bran extract (BPRBE) against *Salmonella* Typhimurium infection, and investigated the mechanisms involved. The results showed that, BPRBE enhanced systemic and cell-autonomous antibacterial activities through the autophagic capture of *Salmonella*, resulting in increased fecal bacteria excretion and decreased bacterial colonization of internal organs. These findings confirm that BPRBE has *in vivo* antimicrobial activities and could be used as a functional antibiotic in the food production industry and medicine. Triclosan (TCS) is a broad-spectrum antibacterial agent widely used in personal care and household cleaning. Shi et al. (2016) investigated the antibacterial properties of TCS and demonstrated that it could indeed induce autophagy via an ERK-dependent pathway, thus enhancing the abilities of macrophages to kill intracellular bacteria. Chiu et al. (2009) synthesized and characterized a new small-molecule agent celled AR-12 and tested its effect on the survival of *Salmonella* Typhimurium in macrophages. The results showed that AR-12 induced autophagy in macrophages and potently inhibited the survival of intracellular *Salmonella* Typhimurium. The ectopic expression of constitutively-activated Akt1 in macrophages partially reversed the AR-12-mediated inhibition of bacterial survival. Furthermore, oral administration of AR-12 reduced hepatic and splenic bacterial burdens, and significantly prolonged the survival of mice infected with *Salmonella* Typhimurium. These findings show that AR-12 can inhibit invasive intracellular bacteria by regulating the level of autophagy, thus promoting the survival of host cells. AR-12 microcapsules, prepared using a novel degradable acetalated dextran (Ac-Dex) biopolymer, further enhanced the clearance of intracellular *Salmonella* and significantly reduced drug toxicity (Hoang et al., 2014). In order to identify small molecules capable of interfering with *Salmonella* survival or replication in macrophages, Nagy et al. (2019) screened the 14,400-compound Maybridge HitFinder v11 library in a high-content Screen for Anti-Infectives. They found that one small molecule, D61, reduced *Salmonella* load both *in vitro* and *in vivo*, through its autophagy-stimulating activity. Qiu et al. (2019) constructed and characterized a polymeric micelle, HAASD-Rapa micelle, which contains a hyaluronan-streptomycin conjugate with an autophagy activator, rapamycin. They found that the HAASD-Rapa micelle could facilitate the host’s intracellular *Salmonella* killing capacity by promoting streptomycin uptake and rapamycin-mediated autophagy activation. Their study also indicated that the combined antibiotic and rapamycin treatment may represent a promising strategy for the eradication of intracellular bacteria. In recent years, cumulative studies have attempted to develop new nanomaterials for the eradication of bacteria. As the single largest reservoir host for *Salmonella*, chickens were used as experimental models to investigate the antibacterial efficacy of iron oxide nanozyme (IONzyme) against *Salmonella* infection in our previous study (Shi et al., 2018). We found that IONzyme enhanced the generation of reactive oxygen species (ROS) to promote the bactericidal effects of acid autophagic vacuoles, thereby inhibiting the survival of invading intracellular *Salmonella* Enteritidis. Collectively, the above findings demonstrate that the search for xenophagy-regulating agents may represent a powerful strategy for combating *Salmonella* infection and maintaining intestinal homeostasis.

**TABLE 2:** Drugs regulate xenophagy to combat *Salmonella* infection.

Drugs	Model systems and results	Refs
Resveratrol	HCT116, HeLa, MEFs, RAW264.7 and THP-1 cells; Zebrafish. Stimulating autophagy and inducing intracellular *Salmonella* clearance.	96
Acacetin	RAW 264.7 and HeLa cells; mice. Restricting intracellular *Salmonella* replication by restoring TFEB-mediated xenophagy.	97
Biochanin A	HeLa, Raw264.7 and THP-1 cells; mice. Enhancing the defense against *Salmonella* infection through AMPK/ULK1/mTOR-mediated autophagy and extracellular traps and reversing SPI-1-dependent macrophage M2 Polarization.	98
1,25-dihydroxy vitamin D3	Caco-2 cells. Promoting the autophagic removal of intracellular *Salmonella* and modulating inflammatory responses to prevent the host from the adverse effects of excessive inflammation.	99
bioprocessed (fermented) rice bran extract	RAW264.7 cells; mice. Enhancing systemic and cell-autonomous antibacterial activities through the autophagic capture of *Salmonella*, resulting in increased fecal bacteria excretion and decreased bacterial colonization of internal organs.	100
Triclosan	HeLa and Raw264.7 cells. Inducing autophagy via an ERK-dependent pathway, thus enhancing the abilities of macrophages to kill intracellular *Salmonella*.	101
AR-12	RAW264.7, J774.1 and THP-1 cells; mice. Inhibiting invasive intracellular *Salmonella* by regulating the level of autophagy, thus promoting the survival of host cells.	102
AR-12 microcapsules	RAW264.7 and primary human monocyte-derived primary macrophages. Enhancing the clearance of intracellular *Salmonella* and significantly reduced drug toxicity.	103
D61	RAW 264.7, mice and human bone marrow-derived primary macrophages, and HeLa cells; mice. Reducing *Salmonella* load both *in vitro* and *in vivo* through its autophagy-stimulating activity	104
HAASD-Rapa micelles	RAW 264.7 and HK-2 cells. Facilitating the host’s intracellular *Salmonella* killing capacity by promoting streptomycin uptake and rapamycin-mediated autophagy activation	105
IONzyme	LMH cells; SPF chicken. Enhancing the generation of reactive oxygen species (ROS) to promote the bactericidal effects of acid autophagic vacuoles, thereby inhibiting the survival of invading intracellular *Salmonella*.	106

## Summary and Future Prospects

In summary, autophagy acts as double-edged sword in the context of intracellular *Salmonella* infection. On the one hand, autophagy is beneficial to the host through the elimination of intracellular *Salmonella*. While, on the other hand, it facilitates intracellular bacterial replication. The prevention and control of *Salmonella* infection has always been considered a major research focus for the scientific community. Using drugs to regulate *Salmonella* autophagy may therefore represent an effective alternative solution to the eradication of intracellular infection caused by this persistent foodborne pathogen.

Although our understanding of *Salmonella* autophagy is ever increasing, many questions remain to be answered. For example, what causes the two contradictory effects of autophagy on intracellular *Salmonella*? Are other, as-yet-unidentified virulent factors or intracellular signals involved in the autophagy of *Salmonella*? How do the different autophagic adaptors interact in the targeting of *Salmonella* for autophagy? Do different serotypes of *Salmonella* affect the autophagic process and the outcome of infection? What are the *Salmonella*-specific autophagy processes and signaling pathways participating in the immune response during infection?

In this era of increasing antibiotic resistance, further study of the mechanisms implicated in bacterial autophagy has important guiding significance in the discovery of novel strategies for the prevention and control of *Salmonella* infection.

## Author Contributions

All authors listed have made a substantial, direct and intellectual contribution to the work, and approved it for publication.

## Conflict of Interest

The authors declare that the research was conducted in the absence of any commercial or financial relationships that could be construed as a potential conflict of interest.
